# Thymic Carcinoma Presenting as Disseminated Intravascular Coagulation

**DOI:** 10.1155/2014/202943

**Published:** 2014-11-12

**Authors:** Mamatha Siricilla, Jawairia Memon, Robert Avery

**Affiliations:** University of Alabama Birmingham Montgomery Internal Medicine, Montgomery, AL 36116, USA

## Abstract

Thymomas and thymic carcinomas are rare tumors, which originate from the epithelial cells of the thymus. We present a case of thymic carcinoma, which presented with DIC as an initial manifestation. DIC improved with corticosteroid treatment and thymic carcinoma was amendable to chemoradiation.

## 1. Introduction

Thymomas and thymic carcinomas originate in the epithelial cells of the thymus. Incidence of thymomas is 1.5 cases per million [1, 2]. Thymic carcinomas are very rare. Thymic carcinomas and thymomas are unique entities [3] and differ in terms of histologic appearance and immunohistochemical genetic features, as well as clinical behavior [4]. Here we present a case, a patient initially diagnosed with disseminated intravascular coagulation (DIC) who was found on further evaluation to also have thymic carcinoma. Although the association of DIC with solid and hematologic malignancies is extensively reported [5, 6], its association with thymic carcinoma is rarely reported. To the best of our knowledge, this is the only case report of thymic carcinoma with DIC as initial and sole manifestation. Another unusual part of our case is the response of DIC to steroids. Review of previous case reports revealed only one case of each thymic carcinoma [7] and thymoma [8], respectively, presenting with DIC and Cushing syndrome and one case of thymic carcinoma presenting with the triad of superior vena cava (SVC) syndrome, cardiac tamponade, and DIC [9].

## 2. Case Report

A 45-year-old woman presented to emergency department with low-grade fever, generalized bruising, ecchymosis, and fatigue of one week duration. Patient denied epistaxis, gum bleeding, hematuria, or vaginal or rectal bleeding. Past medical history was significant only for hypertension. Her family history was negative for malignancy or bleeding diathesis.

Physical examination was positive only for multiple ecchymosis scattered all over her body. There was no epistaxis, mucosal bleeding, organomegaly, or lymphadenopathy. Cardiovascular and lung exams were benign. Patient was neurologically intact and in no acute distress.

**Figure 1:**
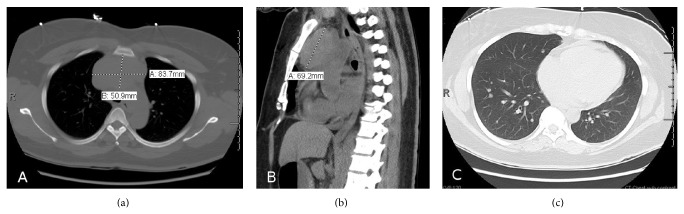
CT scan of the lung showing anterior mediastinal mass measuring 8.4 × 5.1 × 7 cm in anteroposterior (a) and lateral view (b) along with pulmonary nodules (c).

Initial laboratory results are shown in Table 1. Peripheral blood smear showed occasional schistocytes. Direct Coombs test was negative. Chest roentgenogram and computed Tomography (CT) study revealed mediastinal enlargement with a large (8.4 × 5.1 × 7 cm) anterior mediastinal mass and multiple bilateral noncalcified pulmonary nodules ranging from 4 to 8 mm in size (Figure 1).

A diagnosis of DIC was made with the consistent clinical and laboratory findings of a serial trend in elevation of PT, PTT, and D-Dimer and declining in fibrinogen. In the next couple of days patient became overtly symptomatic with oozing of blood from peripheral venous access sites and also developed bleeding from gums.

The patient was treated with packed red blood cells, fresh frozen plasma (FFP), and cryoprecipitate. The patient developed urticarial rash during FFP transfusion and was treated with dexamethasone. After which DIC resolved along with urticarial rash.

**Figure 2:**
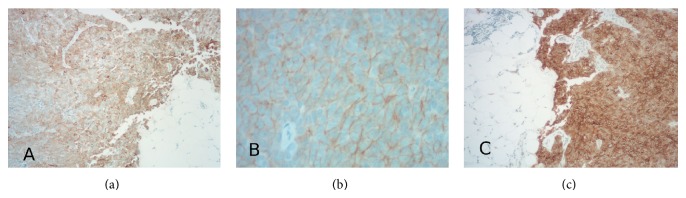
10x Pankeratin positive (a), 40x CD5 Brown-Orange rim around each of the tumor cells (b), and 10x CD117 highlights the tumor and contrasts with the surrounding fat and stromal tissue (c).

Once patient was stable and coagulopathy resolved median sternotomy with biopsy of the anterior mediastinal mass and lung nodules was performed. Pathology revealed the diagnosis to be thymic carcinoma due to the combination of positive pankeratin, CD5, and CD117 (Figures 2(a), 2(b), and 2(c)) and negative staining for chromogranin and only scattered positivity for CD56 and synaptophysin (arguing against neuroendocrine malignancy).

**Figure 3:**
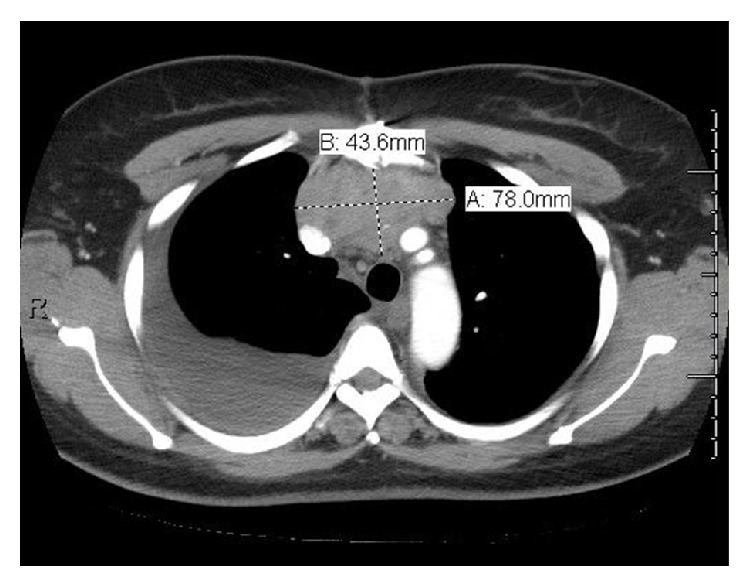
CT scan of the chest after chemotherapy with reduction in size of the tumor.

As per Masaoka-koga staging [10] it was stage III due to lung involvement. Due to unresectable nature of the tumor, neoadjuvant therapy with Cisplatin, doxorubicin, vincristine, and cyclophosphamide (ADOC) was administered. Patient received total of 6 cycles followed by radiation therapy without any major side effects. The size of the tumor on repeat imaging after completion of therapy was found to be 7.8 × 4.4 × 5.5 cm with resolution of the pulmonary nodules (Figure 3). Patient was subsequently evaluated for surgery and deemed not a surgical candidate considering burden of scar tissue in the thorax.

Patient is currently four years from the initial presentation and is doing well, with no symptoms and evidence of recurrence of DIC or progression of the tumor. Her recent CT scans of the chest showed the size of the mass to be 4.3 × 1.9 × 2 cm.

## 3. Discussion

Thymic carcinomas are rare aggressive tumors which frequently metastasize to regional and distant sites and carry a poor prognosis. Although thymomas are locally invasive, it is uncommon to have distant or regional metastases [11, 12]. Five year survival rates are approximately 40% for thymic carcinomas compared to ~78% for thymomas [1, 12–14]. Peak age of incidence is between ages 55 and 65 but cases have been reported to occur at any age. It has no gender predilection. Thoracic metastasis (lung, pericardium, and diaphragm) is common than extrathoracic metastasis; hence tumor node metastasis (TNM classification) does not practically apply for these tumors. These tumors have a tendency to have late recurrence even after complete resection.

Histologic classification of these tumors has been a controversy. World Health Organization (WHO) classification of thymic tumors is based on histologic and morphologic characteristics and has been used widely [15]. Diagnosis of these tumors needs core biopsy, as fine needle aspiration is often not sufficient. Differential diagnosis includes lymphomas, germ cell tumors, neuroendocrine tumors (as it shares some of the markers for neuroendocrine differentiation like synaptophysin and chromogranin) [16], and primary lung malignancies. Staging is done by imaging CT and sometimes Magnetic resonance imaging (MRI) to look for vascular invasion but postoperative surgical staging often carries prognostic value [17].

Treatment of both thymomas and thymic carcinomas is surgical resection. Thymic carcinomas are more aggressive and often present with unresectable mass, so surgical debulking is advised when feasible followed by radiation and chemotherapy depending on the amount of residual tumor. When the tumor is unresectable palliative chemotherapy and/or neoadjuvant therapy can be used [18]. Cisplatin based chemotherapy regimens have been used widely.

Unlike thymic carcinoma, thymomas are associated with paraneoplastic syndromes, the three most common being myasthenia gravis (30–50% of thymomas), pure red cell aplasia, and adult onset hypogammaglobulinemia [19, 20].

Occurrence of DIC with either thymoma or thymic carcinoma is very rare. Review of literature has revealed only 3 such cases where either thymic carcinoma or thymoma was associated with DIC. Of the three cases, one was a case of thymic carcinoma associated with SVC obstruction, cardiac tamponade, and DIC. The patient died within 2 months of initial diagnosis due to recurrent and persistent DIC despite low dose heparin; chemoradiation and surgery aimed to treat thymic carcinoma [9]. The other 2 case reports one of each thymoma [8] and thymic carcinoma [7] presented with DIC and Cushing syndrome succumbed to death secondary to DIC. Our case is unique with responsiveness of DIC to steroids, facilitating successful treatment of thymic carcinoma and survival of the patient. Our case did not have any Cushing syndrome or any other possible cause for DIC such as infection, sepsis, or other autoimmune conditions. The proposed mechanism for the occurrence of DIC in these tumors is release of tissue thromboplastin from necrotic tissue or possibly presence of concurrent cushingoid state which might trigger hypercoagulability (increasing certain clotting factors) and also suppress reticuloendothelial system, thus increased susceptibility to infections ultimately leading to DIC [8]. However the exact pathogenesis underlying this association is not known. It has to be noted that reported cases are rare and both thymomas and thymic carcinomas cases have been seen in association with DIC. Literature review also revealed a case of thymoma in association with autoimmune hemolytic anemia (AIHA) which was successfully treated with corticosteroids [19].

We believe there is an underlying immunologic mechanism associated with thymic carcinoma in causation of DIC. Steroids could be of potential benefit in such situations causing immune suppression.

DIC has a grave prognosis with no proven successful treatment other than supportive care. Mortality can be high as seen in these case reports and often given unstable patient condition and limitation of time it might not be feasible to aim at immediate treatment of the underlying tumor. Often the diagnosis of these tumors can be incidental (as seen in our case), or retrospectively at the time of autopsy. Thus it can be challenging to manage these patients.

## 4. Conclusion

Given the rarity of these tumors and variable presentations, reporting such cases will broaden the clinical spectrum and management options.

## Figures and Tables

**Table 1:** Laboratory values at presentation.

Test	Result	Normal range
White blood cells	13,000/*µ*L	4000–10,000/*µ*L
Hemoglobin	7.0 g/dL	11–15 g/dL
Hematocrit	21%	36–47%
Platelets	68,000/*µ*L	140,000–400,000/*µ*L

Mean corpuscular volume	82 fL	80–100 fL
Partial thromboplastin time	18.3 sec	11–13 sec
Prothrombin time	1.78 sec	0.91–1.10 sec
Fibrinogen	<70 mg/dL	150–350 mg/dL
D-Dimer	29.32 mg/L	0.19–0.49 mg/L
Lactate dehydrogenase	435 U/L	60–100 U/L
Total bilirubin	2.1 mg/dL	0.3–1.2 mg/dL
Indirect bilirubin	1.8 mg/dL	0.3–0.9 mg/dL
